# The developmental phenotype of motor delay in extremely preterm infants following early-life respiratory adversity is influenced by brain dysmaturation in the parietal lobe

**DOI:** 10.1186/s11689-024-09546-9

**Published:** 2024-07-15

**Authors:** Wen-Hao Yu, Chi-Hsiang Chu, Li-Wen Chen, Yung-Chieh Lin, Chia-Lin Koh, Chao-Ching Huang

**Affiliations:** 1 Institute of Clinical Medicine, College of Medicine, National Cheng Kung University, Tainan, Taiwan; 2 Department of Pediatrics, National Cheng Kung University Hospital, College of Medicine, National Cheng Kung University, Tainan, Taiwan; 3 Institute of Statistics, National University of Kaohsiung, Kaohsiung, Taiwan; 4 Department of Occupational Therapy, College of Medicine, National Cheng Kung University, 1 University Road, East District, Tainan City, 70101 Taiwan; 5 Department of Pediatrics, College of Medicine, Taipei Medical University, Taipei, Taiwan; 6 Department of Pediatrics, Shuang Ho Hospital, Taipei Medical University, New Taipei, 23561 Taiwan

**Keywords:** Respiratory support, Altered brain growth, Neurodevelopment, Mediation analysis

## Abstract

**Background:**

Research indicates that preterm infants requiring prolonged mechanical ventilation often exhibit suboptimal neurodevelopment at follow-up, coupled with altered brain development as detected by magnetic resonance imaging (MRI) at term-equivalent age (TEA). However, specific regions of brain dysmaturation and the subsequent neurodevelopmental phenotype following early-life adverse respiratory exposures remain unclear. Additionally, it is uncertain whether brain dysmaturation mediates neurodevelopmental outcomes after respiratory adversity. This study aims to investigate the relationship between early-life adverse respiratory exposures, brain dysmaturation at TEA, and the developmental phenotype observed during follow-up in extremely preterm infants.

**Methods:**

89 infants born < 29 weeks’ gestation from 2019 to 2021 received MRI examinations at TEA for structural and lobe brain volumes, which were adjusted with sex-and-postmenstrual-age expected volumes for volume residuals. Assisted ventilation patterns in the first 8 postnatal weeks were analyzed using kmlShape analyses. Patterns for motor, cognition, and language development were evaluated from corrected age 6 to 12 months using Bayley Scales of Infant Development, third edition. Mediation effects of brain volumes between early-life respiratory exposures and neurodevelopmental phenotypes were adjusted for sex, gestational age, maternal education, and severe brain injury.

**Results:**

Two distinct respiratory trajectories with varying severity were identified: improving (*n* = 35, 39%) and delayed improvement (*n* = 54, 61%). Compared with the improving group, the delayed improvement group exhibited selectively reduced brain volume residuals in the parietal lobe (mean − 4.9 cm^3^, 95% confidence interval − 9.4 to − 0.3) at TEA and lower motor composite scores (− 8.7, − 14.2 to − 3.1) at corrected age 12 months. The association between delayed respiratory improvement and inferior motor performance (total effect − 8.7, − 14.8 to − 3.3) was partially mediated through reduced parietal lobe volume (natural indirect effect − 1.8, − 4.9 to − 0.01), suggesting a mediating effect of 20%.

**Conclusions:**

Early-life adverse respiratory exposure is specifically linked to the parietal lobe dysmaturation and neurodevelopmental phenotype of motor delay at follow-up. Dysmaturation of the parietal lobe serves as a mediator in the connection between respiratory adversity and compromised motor development. Optimizing respiratory critical care may emerge as a potential avenue to mitigate the consequences of altered brain growth and motor developmental delay in this extremely preterm population.

## Background

With the growing survival of extremely preterm infants, the key concern now is reducing neurodevelopmental impairments through enhanced care in the neonatal intensive care unit (NICU). Immediately after birth, preterm infants often require different types of assisted ventilation for weeks or months to reach the targeted range of oxygen saturation due to their immature respiratory systems, before gradually weaning to room air [1]. Early-life use of assisted ventilation is related to bronchopulmonary dysplasia (BPD) [2], and BPD precursors, such as prolonged use of invasive mechanical ventilation (IMV), have been linked to neurodevelopmental impairment at follow-up [2–5]. Therefore, close monitoring of the respiratory pattern is crucial, as it potentially impacts long-term neurodevelopmental outcomes.

Studies have demonstrated that preterm infants who undergo prolonged IMV tend to exhibit lower motor developmental scores [6]. Infants exposed to high cumulative levels of supplemental oxygen are prone to inferior language and cognitive performances [7]. Magnetic resonance imaging (MRI) studies have revealed that alteration of brain development at term-equivalent age (TEA) after adverse exposures in NICUs is associated with neurodevelopmental impairment [8]. For example, infants with prolonged IMV had impaired brainstem development and abnormal white matter maturation [6]. High cumulative supplemental oxygen exposure was associated with a higher degree of white matter injury [9]. Despite these findings, few studies elucidate the intricate links among early-life adverse respiratory exposures, altered brain development at TEA, and subsequent neurodevelopmental phenotype at follow-up.

Our previous study found that preterm infants who experienced a delay in the improvement of their respiratory trajectory within the first eight weeks of life had a higher rate of neurodevelopmental impairment compared to infants whose respiratory trajectory improved more rapidly [5]. However, the specific regions of brain dysmaturation and the subsequent neurodevelopmental phenotype following the early-life adverse respiratory trajectory remain unclear. The current study aims to investigate whether specific brain area dysmaturation mediates neurodevelopmental patterns after respiratory adversity in extremely preterm infants.

## Methods

### Participants

This study prospectively recruited extremely preterm infants who were born less than 29 weeks’ gestation and admitted to a tertiary university hospital from April, 2019 to December, 2021. Brain MRI examinations were performed at TEA, and neurodevelopmental assessments implemented at corrected age 6 and 12 months. This study was approved by the Institutional Review Board for clinical data collection, neuroimaging examinations, and neurodevelopmental assessments.

### Daily types of assisted ventilation

The type of assisted ventilation to keep oxygen saturation in the target range between 88% and 95% by pulse oximetry was recorded daily during the first 8 postnatal weeks. The respiratory support was graded as: 1, Room air or minimal support by oxygen nasal cannula; 2, Moderate support: nasal continuous positive airway pressure or nasal intermittent positive pressure; 3, High support: IMV and IMV-plus, which included high-frequency oscillatory ventilation (HFOV), IMV with inhaled nitric oxide (iNO), or HFOV with iNO [5].

### Neonatal risks

Demographic data and risks in the perinatal and neonatal periods, including maternal educational levels (lower level defined as below college), antenatal steroids use, small for gestational age, and 5-minute Apgar score < 7, were recorded. Major morbidities during hospitalization were documented, which included blood culture-verified sepsis, necrotizing enterocolitis (at least stage II by modified Bell’s staging criteria), grade III/IV intraventricular hemorrhage, cystic periventricular leukomalacia, severe retinopathy of prematurity (defined as ≥ stage II plus) [10], and moderate to severe BPD [11]. Severe brain injury documented by serial ultrasound examinations in the NICU encompassed grade III/IV intraventricular hemorrhage and/or cystic periventricular leukomalacia [12].

### MRI data acquisition and images preprocessing

To avoid motion artifacts, chloral hydrate sedation (50 mg/kg) was used routinely in each preterm infant before MRI examinations that obtained at TEA, using a Philips Ingenia 3T MRI system with 8-channel head coil. T1-weighted images were obtained using the 3D MPRAGE with TR/TE = 9.4/4.5 ms, flip angle = 7º, matrix size = 192 × 192, voxel size = 1.0 mm isotropic resolution, while T2-weighted images were acquired by turbo spin echo echo-planar imaging sequence with TR/TE = 3000/196 ms, flip angle = 90º, matrix size = 224 × 224, slice thickness 1.0 mm, in plane resolution 0.86 × 0.86 mm.

Images were preprocessed and segmented using the Developing Human Connectome Project (dHCP) structural pipeline [13]. T1- and T2-weighted images underwent bias-field correction using ANTs N4 algorithm [14], and brain extraction using the Brain Extraction Tool [15]. Following this, the brain images were segmented into 9 tissue classes using the Draw-EM algorithm [16], and further parcellated into detailed regions based on multi-atlas label fusion approach [13].

By the dHCP pipeline [13], structural brain volumes included intracranial volume (total tissue plus intraventricular and extra-axial cerebrospinal fluid), total brain volume (intracranial volume excluding extra-axial cerebrospinal fluid), total tissue volume (total brain volume excluding intraventricular cerebrospinal fluids), and the individual volume of brainstem, cerebellum, cortical gray matter, cortical white matter, and subcortical gray nuclei. Lobe brain volumes encompassed gray and white matter of the frontal, parietal, temporal, occipital, limbic lobes and insula.

### Brain volume residuals

To accommodate for the effect of gender differences and varied postmenstrual ages (i.e., gestational age plus postnatal age) at MRI examinations, brain volume residuals were calculated by adjusting for sex, and linear and quadratic postmenstrual age at MRI performance [17, 18]. Then, the actual brain volume observed by MRI was compared to this expected volume. A smaller-than-expected brain size resulted in a negative residual, while a larger-than-expected size yielded a positive residual. These residuals provide a standardized measure of brain size that accounts for gender and postmenstrual-related variations.

### Neurodevelopmental phenotypes

Neurodevelopmental patterns were assessed using the Bayley Scales of Infant Development, third edition (BSID-III) at 6 and 12 months of corrected age [19]. The composite scores of cognition, language, and motor domains were derived with a mean score of 100 with one standard deviation of 15. Infants were considered to have delayed development when any of the composite score was less than one standard deviation (< 85) [20]. BSID-III also yielded 5 scaled scores, including cognition, receptive communication, expressive communication, fine motor, and gross motor functions, which were normalized into a mean score of 10 with one standard deviation of 3. Two child psychologists, who were blinded to the infants’ past history and MRI data, conducted the BSID-III assessments.

### Statistics

The respiratory trajectories were characterized using the kmlShape clustering analysis to cluster meaningful groups [21]. kmlShape clustering analysis has been used to analyze time-series and longitudinal data based on their shapes, capturing trajectory heterogeneity within study populations. The number of clusters was determined according to the total within-cluster sum of square and elbow method [22]. Brain volumes, neonatal risk factors, morbidities and outcomes were compared among the different respiratory trajectory groups using chi-square tests or Fisher’s exact tests for categorical variables, and Mann-Whitney U test or analysis of variance for continuous variables, based on the normality assumption. Bonferroni’s correction was applied to address the issue of multiple comparisons of structural and lobe brain volumes.

Residual brain volumes by MRI were calculated from the linear regression model adjusted for gender and postmenstrual age at MRI examinations. Mediation analysis was performed based on the template described by Baron and Kenny [23]. The natural direct effect and natural indirect effect were calculated, and nonparametric bootstrapping procedures with 100 replications were employed to obtain the corresponding 95% confidence intervals (CI) [23, 24]. Sex, gestational age, maternal educational level, and severe brain injury were considered as covariates for adjustment. A *P*-value less than 0.05 (two-tailed test) was considered statistically significant. SPSS version 19 (SPSS Inc., Chicago) and R packages “kmlShape” and “mediation” (R Foundation for Statistical Computing, Vienna) were used for statistical analyses.

## Results

### Early-life respiratory patterns

**Fig. 1:**
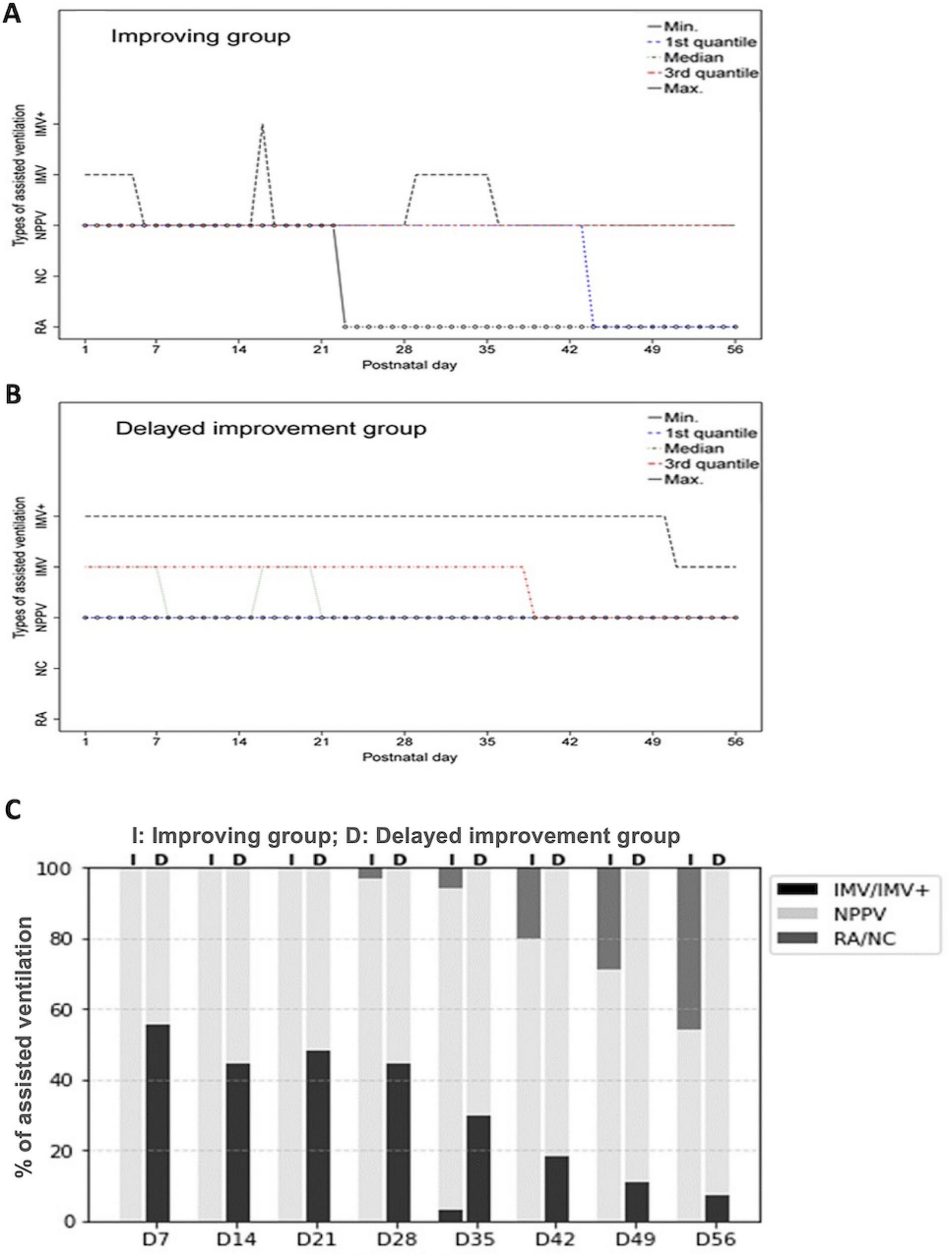
Two distinct respiratory patterns with differential severity. kmlShape clustering analysis categorized respiratory trajectory patterns as improving (*n* = 35, **A**) and delayed improvement (*n* = 54, **B**) based on the daily types of assisted ventilation required for the targeted oxygen saturation levels in the first 8 weeks after birth. Categories of assisted ventilation were invasive mechanical ventilation (IMV), IMV+ (high-frequency oscillatory ventilation (HFOV), IMV with inhaled nitric oxide (iNO) or HFOV with iNO), NPPV that included nasal continuous positive airway pressure or nasal intermittent positive pressure ventilation, and oxygen nasal cannula (NC) or room air (RA). (**C**) The improving group and the delayed improvement group differed in the proportions of infants requiring IMV/IMV+, NPPV, or NC/RA from postnatal day 7 to day 56, showing a transition from IMV/IMV + to NPPV in the delayed improvement group, and a transition from NPPV to NC/RA in the improving group

During the study period, 89 extremely preterm infants with median gestational age of 26 weeks (interquartile range 3 weeks) received MRI examinations at TEA (median postmenstrual age 42 weeks, interquartile range 7 weeks). Utilizing the daily respiratory data of these infants in the first 8 postnatal weeks, the kmlShape analysis discerned two distinct patterns with differential severity: improving (Fig. 1A, *n* = 35, 39%) and delayed improvement (Fig. 1B, *n* = 54, 61%).

By postnatal days 7, 14, 21, 28 and 35, the delayed improvement group had a significantly higher proportion of infants requiring invasive respiratory support (IMV or IMV+) compared to the improving group (all *P* < 0.001). At later time points (days 42, 49, and 56), the delayed improvement group still had a significantly lower proportion of infants who had transitioned to oxygen nasal cannula or room air compared to the improving group (all *P* < 0.001) (Fig. 1C).

### Risk differences between the two respiratory pattern groups

The two groups were similar in terms of sex, maternal educational levels, and the presence of severe brain injury (Table 1). The delayed improvement group was significantly lower in gestational age and birth body weight, and had a higher proportion of infants with 5-minute Apgar scores less than 7 and moderate to severe BPD compared to the improving group (all *P* < 0.001).

**Table 1:** Neonatal risks, morbidities, and neurodevelopmental outcomes of the improving and delayed improvement respiratory trajectory groups

	Respiratory Trajectories		
	Improving	Delayed improvement	
**Neonatal characteristics**	*n* = 35	*n* = 54	*P*-value
Gestational age (wks), mean (SD)	26.9 ± 1.1	24.9 ± 1.9	**< 0.001**
Birth bodyweight (gram), mean (SD)	972 ± 206	757 ± 213	**< 0.001**
Male, n (%)	21 (60)	32 (59)	0.95
Lower maternal education^1^, n (%)	15 (43)	19 (35)	0.47
Small for gestational age, n (%)	1 (3)	6 (11)	0.24
Antenatal steroids, n (%)	35 (100)	53 (98)	1.00
5-minute Apgar score < 7, n (%)	4 (11)	27 (50)	**< 0.001**
Sepsis/severe NEC, n (%)	7 (20)	20 (37)	0.10
Severe brain injury^2^, n (%)	2 (6)	11 (20)	0.07
**Postmenstrual age 36 weeks**			
Moderate/severe BPD^3^, n (%)	6 (17)	36 (67)	**< 0.001**
Severe ROP^4^, n (%)	3 (9)	14 (26)	0.05
**Neurodevelopment outcomes by BSID III** ^5^	*n* = 33	*n* = 52	*P*-value
**Corrected age 6 months**, **median (Quartile 1, Quartile 3)**			
Cognitive composite score	95 (95, 103)	95 (85, 100)	0.06
Language composite score	97 (94, 103)	94 (94, 100)	0.06
Motor composite score	94 (88, 105)	91 (83, 100)	**0.03**
**Corrected age 12 months**, **median (Quartile 1, Quartile 3)**			
Cognitive composite score	100 (100, 108)	100 (95, 109)	0.14
Language composite score	97 (94, 103)	94 (89, 97)	**0.005**
Motor composite score	94 (91, 99)	90 (85, 94)	**0.001**

NEC: necrotizing enterocolitis; BPD: bronchopulmonary dysplasia, ROP: retinopathy of prematurity

^1^Lower maternal education defined as below the college level

^2^Severe brain injury included cystic periventricular leukomalacia and/or grade III/IV intraventricular hemorrhage

^3^Moderate/severe BPD defined by ≥ 28 days of supplemental oxygen and in need of oxygen supply or positive pressure ventilation at 36 weeks’ post-menstrual age

^4^Severe ROP: retinopathy of prematurity defined as ≥ stage II plus

^5^BSID-III: the Bayley Scales of Infant Development, third edition

### Neurodevelopmental differences between the two respiratory pattern groups

Overall, 85 (96%) of the 89 infants had neurodevelopmental assessments at 6 and 12 months of corrected age. Compared to the improving group, the delayed improvement group had significantly lower motor composite scores at corrected age 6 months (*P* = 0.03), and lower language and motor composite scores at corrected age 12 months (both *P* < 0.01) (Table 1).

### Association between delayed improvement in respiratory pattern and brain volume residuals

After excluding infants with MRI of significant motion artifacts which were difficult for segmentation, 79 (89%) of the 89 infants were available for neuroimaging analyses. Before adjusting for sex, and linear and quadratic postmenstrual ages at MRI, all the structural and lobe brain volumes listed in Table 2 were comparable between two respiratory trajectory groups (Table 2).

**Table 2:** Differences in brain volumes at term equivalent age between the improving and delayed improvement groups

	Respiratory Trajectories			
	Improving	Delayed improvement		
	*n* = 31	*n* = 48	*P*-value	
**Demographics**				
Male, n (%)	19 (61.3)	27 (56.3)	0.66	
PMA at MRI, wks, mean (SD)	41.5 (3.7)	42.5 (4.7)	0.34	
**Structural brain volume, cm**^**3**^, **mean (SD)**			*P*-value	Adjusted *P*-value*
Intracranial volume (ICV)	484.3 (97.4)	451.5 (111.0)	0.18	1.00
Total brain volume (TBV)	385.9 (71.8)	357.2 (88.3)	0.13	1.00
Total tissue volume (TTV)	378.1 (69.5)	347.6 (86.9)	0.10	1.00
TTV minus Brainstem and cerebellum	344.9 (60.5)	317.2 (75.0)	0.09	1.00
TTV/ICV ratio	0.78 (0.03)	0.77 (0.04)	0.18	
TTV minus/ICV ratio	0.72 (0.03)	0.71 (0.04)	0.27	
Brainstem	6.6 (1.0)	6.2 (1.3)	0.15	1.00
Cerebellum	26.7 (8.6)	24.4 (11.4)	0.34	1.00
Extra-axial cerebrospinal fluid	98.4 (31.4)	94.4 (28.3)	0.56	1.00
Ventricles	7.7 (3.3)	9.1 (6.2)	0.25	1.00
Cortical gray matter	162.0 (40.4)	149.7 (48.1)	0.24	1.00
Cortical white matter	162.1 (19.3)	147.9 (28.0)	**0.009**	0.15
Subcortical gray nuclei	22.8 (3.8)	21.9 (4.3)	0.34	1.00
**Lobe brain volumes**				
Frontal lobe	117.0 (20.7)	106.4 (25.1)	0.05	0.85
Parietal lobe	77.8 (14.9)	70.8 (18.6)	0.09	1.00
Temporal lobe	49.6 (8.4)	48.0 (13.2)	0.52	1.00
Occipital lobe	48.0 (9.4)	45.3 (10.9)	0.26	1.00
Insula	8.9 (1.3)	8.3 (2.9)	0.30	1.00
Limbic lobe	20.9 (3.8)	19.4 (4.5)	0.14	1.00

PMA: Postmenstrual age (i.e., gestational age plus postnatal age); MRI: magnetic resonance imaging

ICV: Intracranial volume, all brain gray matter tissue + white matter tissue + ventricles + extra-axial cerebrospinal fluid

TBV: Total brain volume, all brain gray matter tissue + white matter tissue + ventricles

TTV: Total tissue volume, all brain gray matter tissue + white matter tissue

* Bonferroni’s correction

Brain volume residuals were computed to show the variance between the individual’s actual brain volume and the expected volume based on the regression model adjusted for sex and the postmenstrual ages at MRI performance. The delayed improvement group had significantly lower brain volume residuals in most brain regions of interest, including total tissue volume, cerebellum, cortical gray matter, cortical white matter, subcortical gray nuclei, frontal lobe, parietal lobe, temporal lobe, and insula compared with the improving group (all adjusted *P* < 0.05) (Table 3). Using the improving group as reference, the delayed improvement group exhibited a significant mean reduction of 4.85 cm^3^ in the parietal lobe volume residuals after adjusting for the covariates (*P* = 0.04) (Table 4).

**Table 3:** Differences in brain volume residuals at term equivalent age between the two respiratory trajectory groups

	Respiratory Trajectories			
	Improving	Delayed improvement		
	*n* = 31	*n* = 48	*P-*value	Adjusted *P*-value*
**Structural brain volume residuals**^**1**^, **cm**^**3**^, **mean (SD)**				
Total tissue volume^2^	22.3 (29.6)	−14.4 (49.7)	**< 0.001**	**0.004**
Total tissue volume minus Brainstem and cerebellum	19.7 (26.8)	−12.7 (43.8)	**< 0.001**	**0.007**
Brainstem	0.3 (0.6)	−0.2 (0.8)	**0.004**	0.077
Cerebellum^3^	0.1 (0.2)	−0.1 (0.3)	**0.001**	**0.009**
Cortical gray matter^3^	0.1 (0.1)	−0.0 (0.2)	**0.001**	**0.023**
Cortical white matter	9.7 (12.5)	−6.3 (21.5)	**< 0.001**	**0.004**
Subcortical gray nuclei	1.1 (1.8)	−0.7 (3.0)	**0.003**	**0.027**
**Lobe brain volume residuals**^**1**^, **cm**^**3**^, **mean (SD)**				
Frontal lobe	7.5 (10.1)	−4.9 (16.0)	**< 0.001**	**0.006**
Parietal lobe	4.8 (7.0)	−3.1 (10.8)	**< 0.001**	**0.017**
Temporal lobe^3^	0.1 (0.1)	−0.0 (0.1)	**< 0.001**	**0.027**
Occipital lobe	2.4 (4.9)	−1.6 (6.7)	**0.005**	0.082
Insula	0.6 (0.9)	−0.4 (1.2)	**< 0.001**	**< 0.001**
Limbic lobe	0.8 (2.4)	−0.5 (2.6)	**0.036**	0.466

^1^Brain volume residuals were calculated after adjusting for sex and linear and quadratic postmenstrual ages (i.e., gestational age plus postnatal age) at MRI.

Brain volume residuals: ${R}_{i}={Y}_{i}-{\widehat{Y}}_{i},$

where ${R}_{i}$ is Brain volume residual, ${Y}_{i}$ is Brain volume, and ${\widehat{Y}}_{i}={b}_{0}+{b}_{1}\times \text{Male}+{b}_{2}\times \text{P}\text{M}\text{A}+{b}_{2}\times {\text{P}\text{M}\text{A}}^{2}$

where ${b}_{i}$’s are corresponding estimated coefficients by calculating from the linear regression model

* Bonferroni’s correction

**Table 4:** Associations between early-life respiratory patterns and brain volume residuals at term equivalent age

Structural brain volume residuals^1^	β	95% CI	Adjusted *P*-value
Total tissue volume^2^	−17.6	(− 36.8, 1.6)	0.07
Total tissue volume minus Brainstem and cerebellum	−15.4	(− 32.5, 1.8)	0.08
Brainstem	−0.3	(− 0.7, 0.03)	0.07
Cerebellum^3^	−0.1	(− 0.2, 0.03)	0.15
Cortical gray matter^3^	−0.05	(− 0.1, 0.01)	0.09
Cortical white matter	−7.3	(− 15.8, 1.3)	0.09
Subcortical gray nuclei	−1.1	(− 2.2, 0.1)	0.07
**Lobe brain volume residuals** ^**1**^			
Frontal lobe	−4.5	(− 10.6, 1.7)	0.15
Parietal lobe	−4.9	(− 9.4, − 0.3)	**0.04**
Temporal lobe^3^	−0.04	(− 0.1, 0.01)	0.11
Occipital lobe	−2.5	(− 5.4, 0.5)	0.10
Insula	−0.4	(− 0.9, 0.1)	0.13
Limbic lobe	−0.4	(− 1.6, 0.8)	0.54

Infants with improving respiratory trajectory as reference. Covariates used in the adjusted mode included gestational age, maternal educational level, and severe brain injury

^1^Brain volume residuals were calculated after adjusting for sex and linear and quadratic postmenstrual ages (i.e., gestational age plus postnatal age) at MRI.

Brain volume residuals: ${R}_{i}={Y}_{i}-{\widehat{Y}}_{i},$

where ${R}_{i}$ is Brain volume residual, ${Y}_{i}$ is Brain volume, and ${\widehat{Y}}_{i}={b}_{0}+{b}_{1}\times \text{Male}+{b}_{2}\times \text{P}\text{M}\text{A}+{b}_{2}\times {\text{P}\text{M}\text{A}}^{2}$

where ${b}_{i}$’s are corresponding estimated coefficients by calculating from the linear regression model

^2^Total tissue volume: all brain gray matter tissue + white matter tissue

^3^Log-transformation was applied due to the non-normal distribution of brain volume residuals

### Associations of respiratory patterns and brain volume residuals with neurodevelopment

Using the improving group as reference, the delayed improvement group was significantly associated with lower motor scores at corrected age 12 months after adjustment (*β* = −8.7, 95% CI − 14.2 to − 3.1) (Table 5). After adjusting for the covariates, the reduction of brain volume residuals in the parietal lobe was negatively associated with the cognitive (*β* = −0.4, 95% CI − 0.7 to − 0.2), language (*β* = −0.3, 95% CI − 0.5 to − 0.1), and motor (*β* = −0.5, 95% CI − 0.7 to − 0.2) scores (Table 5).

**Table 5:** Associations of early-life respiratory patterns and parietal lobe volume residuals with neurodevelopmental outcomes

	Neurodevelopmental outcomes at corrected age 12 months, β (95% CI)		
	Cognitive composite score	Language composite score	Motor composite score
**Delayed improvement** **in respiratory trajectory**			
Crude	−3.2 (− 8.3, 1.9)	−3.9 (− 7.5, − 0.3)^*^	−7.6 (− 12.8, − 2.4)^*^
Adjusted	−3.6 (− 9.3, 2.1)	−3.0 (− 6.9, 0.8)	−8.7 (− 14.2, − 3.1)^*^
**Reduction of parietal lobe volume residuals**			
Crude	−0.3 (− 0.6, − 0.1)^*^	−0.3 (− 0.5, − 0.2)^*^	−0.5 (− 0.7, − 0.2)^*^
Adjusted	−0.4 (− 0.7, − 0.2)^*^	−0.3 (− 0.5, − 0.1)^*^	−0.5 (− 0.7, − 0.2)^*^

Infants with improving respiratory trajectory as reference. Covariates used in the adjusted mode included gender, gestational age, maternal educational level, and severe brain injury

^*^P < 0.05

### Causal mediation analyses

After adjusting for the covariates, the effect of early-life delayed improvement in respiratory trajectory had a substantial association with the motor score, showing a total effect of − 8.7 (95% CI − 14.8 to − 3.3, *P* < 0.001). The total effect could be represented with a natural direct effect of − 6.9 (95% CI − 12.2 to − 2.3, *P* < 0.001), signifying the association between the delayed respiratory trajectory and the motor score that was not mediated by parietal lobe brain volumes. Meanwhile, the natural indirect effect, representing the portion of the association mediated through parietal lobe brain volumes, was − 1.8 (95% CI − 4.9 to − 0.01, *P* < 0.05) (Table 6**)**. The mediating effect of the parietal lobe brain volumes accounted for approximately 20% (*P <* 0.05) of association between delayed respiratory trajectory and motor score.

**Table 6:** Causal mediation analysis. Mediation effects of the parietal lobe residual brain volumes at term equivalent age on the association between early-life adverse respiratory pattern and neurodevelopmental outcomes at corrected age 12 months

	Motor composite score	Gross motor scaled score	Fine motor scaled score
	Estimate (95% CI), *P*-value		
**Total effect**	−8.7 (− 14.8, − 3.3), *P* < 0.001	−1.6 (− 2.9, − 0.5), *P* = 0.002	−1.2 (− 2.3, − 0.4), *P* = 0.002
**Natural direct effect** ^**1**^	−6.9 (− 12.2, − 2.3), *P* < 0.001	−1.3 (− 2.5, − 0.3), *P* = 0.01	−1.0 (− 1.8, − 0.2), *P* = 0.006
**Natural indirect effect** ^**2**^	−1.8 (− 4.9, − 0.01), *P* < 0.05	−0.3 (− 0.8, 0.00), *P* < 0.05	−0.3 (− 0.8, 0.03), *P* = 0.10
**Proportion mediated**	0.20 (0.01, 0.55), *P* < 0.05	0.19 (0.00, 0.67), *P* = 0.05	0.22 (− 0.04, 0.69), *P* = 0.10

Covariates in the adjusted mode included gender, gestational age, maternal educational level, severe brain injury

Nonparametric bootstrap was used for the causal mediation analysis:

Exposure- delayed improvement in respiratory trajectory; Mediator- residual brain volumes of parietal lobe; Outcome- neurodevelopment by BSID-III.

^1^Natural direct effect: the effect of exposure on the outcome in the absence of the mediator

^2^Natural indirect effect: the effect of exposure on the outcome that works through the mediator

## Discussion

**Fig. 2:**
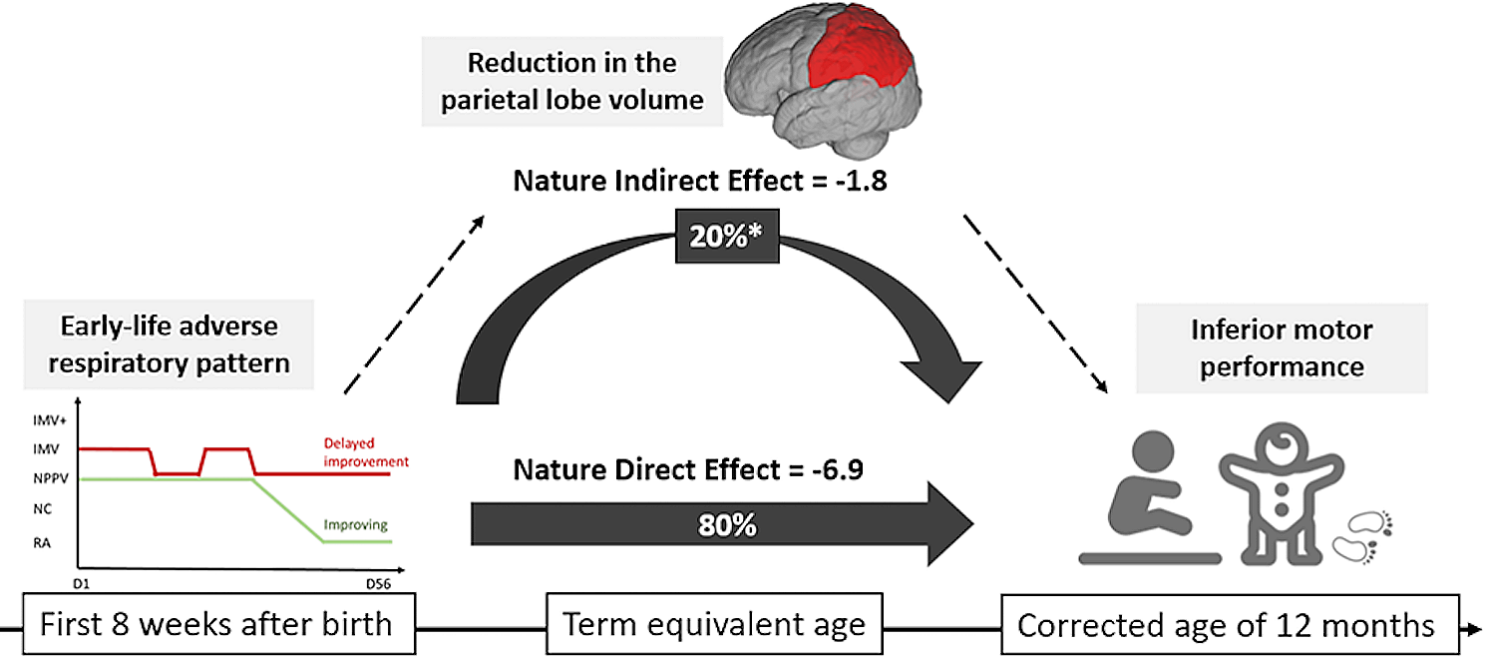
Parietal lobe volume partially mediates the effect of respiratory exposure on motor performance. Reduced brain volume residuals in the parietal lobe at term equivalent age account for 20% of the association effect between early-life delayed improvement of respiratory trajectory and motor composite scores at corrected age 12 months. *Proportion of mediation

This study elucidated the intricate interconnections among the sequential events occurring at three time points: adverse respiratory exposure during the first 8 postnatal weeks, brain dysmaturation by TEA, and developmental motor delay at corrected age 12 months. We showed that extremely preterm infants who followed the delayed improvement of respiratory trajectory exhibited selectively reduced residual parietal lobe volumes at TEA, and lower motor performance at follow-up compared to infants who followed the improving respiratory trajectory. Causal mediation analyses revealed that part of the association between delayed respiratory improvement and inferior motor performance was mediated through reduction of the parietal lobe volume (Fig. 2).

The duration of IMV in the NICU has been recognized as a risk for neurodevelopmental impairment in preterm infants [4, 25, 26]. Guillot et al. reported that early-life prolonged IMV was associated with lower motor scores at preschool age [6]. Vliegenthart et al. found the IMV duration that associated with increased risk for neurodevelopmental impairment was mainly driven by the lower motor score at corrected age 24 months [4]. The pattern of assisted ventilation varies not only by gestational age but also by individual differences among infants of the same gestational age [1, 5, 8]. Instead of solely relying on the IMV duration, our study incorporated longitudinal sequence of assisted ventilation, i.e., the trajectory from IMV to room air, to identify the distinct infants who followed a high-risk respiratory trajectory associated with a specific type of neurodevelopmental delay. Our previous study showed that infants who followed the adverse respiratory trajectory had a higher rate of neurodevelopment impairment [5]. Current study further demonstrated that the adverse respiratory trajectory specifically associated with lower motor performance.

Brain dysmaturation or injury identified by MRI at TEA has been used as a predictor of neurodevelopmental impairments in preterm infants. There appears to be specific regions in the immature brain that are most vulnerable to early-life adverse exposures. For example, BPD was a significant predictor of delayed brain maturation [27], and infants with BPD exhibited significantly impaired development in the white matter and cerebellum compared to infants without BPD [28]. Prolonged IMV has also been linked to adverse brain development, such as impaired brainstem development and abnormal white matter maturation [6], as well as alterations in insula, parahippocampus, and left temporal area [29]. By examining the lobe brain volumes, our work emphasized the selective vulnerability of parietal lobe following early-life adverse respiratory exposures.

The parietal lobe plays a critical role in an array of functions including sensorimotor integration, preliminary motor planning, spatial attention, and advanced cognitive activities [30]. In preterm infants without significant brain injury, studies have shown that brain tissue volumes in the parietal lobe at TEA were associated with early motor behavior [31], and the gyrification index of the left parietal lobe predicted gross motor outcome [32]. In addition, decreased cortical complexity in the medial parietal cortices was linked with reduced intelligence quotients of preterm-birth adults, mediating the association between cognitive development at age 20 months and intelligence in adulthood [33]. Our work showed that brain volume residuals in the parietal lobe were linked to cognitive, language, and motor developmental outcomes. These findings were in line with the study from Schneider et al. revealing that an association of the growth of brain volumes at TEA predicted psychomotor outcome at 18 months’ corrected age [34].

Few studies have delineated the mediation effect of MRI structural abnormality at TEA in the association between early-life adverse events and neurodevelopmental outcome in preterm infants. Logan et al. revealed that biomarkers of cortical maturation at TEA mediated a substantial portion of the risks conveyed by perinatal illness severity on neurodevelopmental outcomes [35]. Schneider et al. demonstrated that brain growth at TEA served as an intermediary between nutrition intakes in the first 2 weeks of life and neurodevelopment at follow-up [34]. Our study found selective reduction in the parietal lobe volume at TEA that mediated 20% of the risks conveyed by adverse respiratory exposure to inferior motor outcome. While the parietal lobe dysmaturation mediates only a portion of the association, there are likely additional biological factors or pathways that exert influences on motor performance in infants who experienced early-life adverse respiratory exposure. The complex pathways linking adverse respiratory pattern, altered brain growth, and neurodevelopmental outcomes in preterm infants are still not completely understood [26]. Factors that play roles in the lung-brain axis of prematurity include inflammation, altered nutrient intakes, microbiota dysbiosis, and disrupted metabolomics [26, 34, 36, 37].

This study has some limitations. Instead of using NDI as outcome measures at a specific time point such as corrected age 24 months, we used the composite scores of cognition, language, and motor domains derived by the BSID-III to depict the cognition, motor, and language performance phenotypes at 6 and 12 months, respectively, which may be more informative in association with early-life respiratory patterns, and the regional brain volume changes measured by MRI at TEA [38, 39]. The marginal significance of the proportion mediated by the parietal lobe on gross motor scaled scores may be constrained by the number of infants included in the analysis. A larger sample size might provide more robust results. This study used volumetric MRI to highlight the mediating role of the parietal lobe in motor performance outcomes following an adverse respiratory trajectory. Whether altered structural and functional connectivity also underlined delayed motor performance remain to be elucidated [40]. Our study underscores the importance of multicenter longitudinal research with larger cohorts, which can be used to characterize the respiratory patterns that may early predict which infants in the two respiratory groups will be transitioned to cannula or room air more quickly.

It is important to delineate the sequential relationship between early-life adverse respiratory exposures, dysmaturation of specific brain areas, and neurodevelopmental phenotypic outcomes. Optimizing respiratory critical care practices—including antenatal use of steroids, delayed cord clamping, early nasal CPAP, minimally invasive surfactant therapy to reduce IMV use, and standardized weaning strategies for early withdrawal of invasive respiratory support—may be crucial for improving neurodevelopmental outcomes in preterm infants [41–46]. Furthermore, our findings suggest early identification of infants who may follow the adverse respiratory trajectory within days after birth, by medical alertness of the cumulative IMV duration, is possible for timely respiratory intervention.

## Conclusions

Early-life adverse respiratory exposure is specifically linked to the parietal lobe dysmaturation and neurodevelopmental phenotype of motor delay. Dysmaturation of the parietal lobe at TEA serves as a mediator in the connection between early-life respiratory adversity and compromised motor development at follow-up. Optimizing respiratory critical care may emerge as a potential avenue to mitigate the consequences of altered brain growth and motor developmental delay in this extremely preterm population.

## Data Availability

The raw data collected for this study was from the National Cheng Kung University Hospital, and the authors did not obtain permission to publicly share these data. Requests for access to the data may be made to the Institutional Review Board of the University Hospital.

## Abbreviations

BPD: Bronchopulmonary Dysplasia
BSID-III: Bayley Scales of Infant Development, third edition
CI: Confidence Interval
dHCP: Developing Human Connectome Project
HFOV: High-Frequency Oscillatory Ventilation
ICV: Intracranial Volume
IMV: Invasive Mechanical Ventilation
iNO: Inhaled Nitric Oxide
MRI: Magnetic Resonance Imaging
NICU: Neonatal Intensive Care Unit
NPPV: Nasal Intermittent Positive Pressure
TBV: Total Brain Volume
TEA: Term-Equivalent Age
TTV: Total Tissue Volume
